# *Melicope oppenheimeri*, section Pelea (Rutaceae), a new species from West Maui, Hawaiian Islands: with notes on its ecology, conservation, and phylogenetic placement

**DOI:** 10.3897/phytokeys.69.8844

**Published:** 2016-08-25

**Authors:** Kennetah R. Wood, Marc S. Appelhans, Warren L. Wagner

**Affiliations:** 1 National Tropical Botanical Garden, 3530 Papalina Road, Kalaheo, HI 96741, USA; 2 Department of Systematic Botany, Albrecht-von-Haller Institute of Plant Sciences, University of Göttingen, Untere Karspüle 2, 37073 Göttingen, Germany; 3 Department of Botany, Smithsonian Institution, PO Box 37012, Washington, DC 20013-7012, USA

**Keywords:** Rutaceae, Melicope, Melicope section Pelea, new species, Hawaiian Islands, West Maui, Critically Endangered

## Abstract

*Melicope oppenheimeri* K.R. Wood, Appelhans & W.L. Wagner (section Pelea (A. Gray) Hook. f., Rutaceae), a rare endemic tree from West Maui, Hawaiian Islands, is described and illustrated with notes on its ecology, conservation, and phylogenetic placement. The new species differs from Hawaiian congeners by its carpels basally connate 1/5, narrowed into a strongly reflexed beak 10–15 mm long. It also differs in a combination of leaves with 7–10 pair of secondary veins; cymes to 3 cm long; peduncles 5–6.5 mm long; flowers perfect; capsules 4–9 × 40–52 mm; and a densely appressed short-sericeous ovary. *Melicope oppenheimeri* is known only from an isolated cliff-base plateau in upper Waihe‘e Valley, West Maui. Its discovery brings the number of recognized *Melicope* J.R. Forst. & G. Forst. species in the Hawaiian Islands to 49. A table is included indicating the conservation status of Hawaiian *Melicope* and *Platydesma* H. Mann., which is nested within Melicope sect. Pelea.

*Melicope oppenheimeri* falls into the IUCN Critically Endangered (CR) Red List category.

Critically Endangered

## Introduction

*Melicope* J.R. Forst. & G. Forst. is the largest genus of the *Citrus* family (Rutaceae) and consists of ca. 235 species of shrubs and trees (Hartley 2001, Appelhans et al. 2014a). The distribution of *Melicope* ranges from the Malagasy and Indo-Himalayan regions in the east to the Hawaiian and Marquesan Islands in the west and from Nepal, southern China, Taiwan and the Japanese Ogasawara Islands in the north to New Zealand and Australia in the south (Hartley 2001). Ongoing phylogenetic studies of *Melicope* and closely related genera indicate the need for revisionary work in this group with several of the related small genera belonging in *Melicope* in order for it to be monophyletic (Harbaugh et al. 2009, Appelhans et al. 2014b). In the most recent systematic revision by Hartley (2001) *Melicope* was subdivided into four sections: *Lepta* (Lour.) T.G. Hartley; *Melicope*; *Pelea* (A. Gray) Hook. f.; and *Vitiflorae* T.G. Hartley. Only section Lepta proved to be a monophyletic group in a recent molecular study (Appelhans et al. 2014b). The currently known 48 endemic Hawaiian species are all members of sect. Pelea, which consists of 85 species almost exclusively restricted to the Australasian–Outer Melanesian–Pacific region. Within sect. Pelea, only the Hawaiian group proved to be monophyletic (Appelhans et al. 2014c). The Hawaiian endemic species all have unifoliolate leaves; plants presumably hermaphroditic or dioecious; carpels that range from being basally connate to fully connate, and with variations in exocarp and endocarp that contrast from glabrous to pubescent. Hartley (2001) inferred that *Melicope* was present in eastern Gondwanaland before about 96 Ma and states that the Hawaiian species represent a radiation that most likely traces back to a single colonization of the archipelago by a species from sect. Pelea. While Hartley’s age estimate of *Melicope* is doubtful since the oldest fossils of the whole family date to the Late Cretaceous (Knobloch and Mai 1986, Gregor 1989) and molecular dating studies estimated its origin in the Oligocene or Miocene (Muellner et al. 2007, Appelhans et al. 2012), his suggestion for a single colonization of the Hawaiian archipelago was supported by molecular phylogenetic analyses (Harbaugh et al. 2009, Appelhans et al. 2014b, c). The most recent molecular phylogenetic studies also indicate that the Hawaiian genus *Platydesma* H. Mann is nested within Melicope sect. Pelea and that the seven known Marquesan *Melicope* endemics are a closely related group resultant from two independent colonization events from Hawaiian *Melicope* (Harbaugh et al. 2009, Appelhans et al. 2014c).

## Methods

All measurements and descriptions were taken from dried herbarium specimens or from notes made in the field and are presented in the descriptions as follows: length × width, followed by units of measurement (mm or cm).

## Taxonomic treatment

### Melicope oppenheimeri

Taxon classificationPlantaeSapindalesRutaceae

K.R. Wood, Appelhans & W.L. Wagner sp. nov.

urn:lsid:ipni.org:names:60472944-2

Figs 1 , 4 , 5

#### Diagnosis.

Differs from Hawaiian congeners by its combination of leaves having 7–10 pair of secondary veins; cymes to 3 cm long; peduncles 5–6.5 mm long; flowers perfect; carpels basally connate 1/5, narrowed into a strongly reflexed beak 10–15 mm long, capsules 4–9 × 40–52 mm; and ovary appressed densely short-sericeous.

#### Type.

United States of America. Hawaiian Islands, West Maui: Wailuku District, Waihe‘e Valley, Metrosideros polymorpha var. glaberrima-*Cheirodendron trigynum* wet forest, 20°54.15'N; 156°33.95'W, 770 m elev., 12 Sep 2006, *Hank Oppenheimer & Jill Miller H90609* (holotype: PTBG-070667; isotypes: BISH, US)

#### Description.

***Trees*** 3–4 m tall, bark medium brown, young branchlets light brown, glabrate, 3–6 mm wide in third internode, terminal branchlets yellowish brown tomentose with a waxy scurf. ***Leaves*** opposite, unifoliolate, coriaceous, the blade obovate to broadly elliptic, occasionally orbicular, 5–17.5 × 3.5–8.5 cm, the margin entire, the base rounded to obtuse, the apex rounded, obtuse to acute, or emarginate, secondary veins usually 7–10 pairs, connected by a moderately arched vein 2–11 mm from margin with higher order venation reticulate, both surfaces glabrous, occasionally glabrate along midrib of abaxial surface, young leaves glabrate to sparsely puberulent on lower surface; petiole shallowly canaliculate, 10–30 × 1–3 mm at middle, glabrate. ***Flowers***: perfect, 3–5 in axillary cymes up to 3 cm long, peduncles 5–6.5 mm long, sparsely short-puberulent, pedicles 5–8 mm, short-puberulent, bracteoles 1.5–3 mm long, sepals broadly ovate, tip short acuminate, externally sparsely short-puberulent, glabrous within, 3 × 2.5–2.8 mm, connate basally 1/4 to 1/3 of length; petals tinged purple, narrowly ovate, lanceolate, 6–8 × 2.5–3 mm, glabrous internally and externally, tips recurved, nectary disk with sparsely scattered hairs; ovary densely appressed short-sericeous; style ca. 1.7 mm long, with finely appressed hairs; stigma capitate, four lobed, glabrous; stamens 8, filaments glabrous, the antesepalous ones 5–7 mm long, antepetalous ones 4–5 mm long, all with pollen. ***Capsules*** purple tinged when fresh, 4–9 × 40–52 mm, carpels connate basally for ca. 1/5 their length, narrowed into a strongly reflexed beak 10–15 mm long, exocarp glabrate with few hairs widely spaced over surface, endocarp sparsely to evenly puberulent. ***Seeds*** 2 per carpel, ovoid, 5–9 mm long.

#### Phenology.

To date, *Melicope oppenheimeri* has been observed with flower buds in January and August, with flowers at anthesis during September, and with fruit during January, February, May, August, September, and November.

#### Etymology.

We are pleased to name this new species in honor of Hank Oppenheimer, botanist with the Maui Nui Plant Extinction Prevention Program, who collected the type specimen and has made many valuable contributions to the understanding and conservation of the Hawaiian flora.

#### Specimens examined.

**United States. Hawaiian Islands, West Maui**: Wailuku District, Waihe‘e Valley, south side, below and north of Kahoolewa Ridge, 20°54.15'N; 156°33.95'W, 770 m elev., 8 Aug 1998, *Wood & Perlman 7408* (BISH, PTBG, US); loc. cit., 9 Aug 1998, *Perlman & Wood 16,338* (CANB, PTBG); loc. cit., 10 AUG 1998, *Wood & Perlman 7419* (BISH, PTBG, US); loc. cit., 15 Feb 2005, *Oppenheimer & Hansen H20,505* (BISH, PTBG, US); loc. cit., 19 May 2009, *Perlman & Oppenheimer 21,642* (PTBG).

The following couplets can be inserted into the existing key to Hawaiian *Melicope* (treated as *Pelea*) by Stone, Wagner, and Herbst (in Wagner et al. 1999, pp. 1179–1182) to accommodate *Melicope oppenheimeri*.

**Table:** 

19(18)	Exocarp sparsely to densely puberulent or tomentose, at least toward base or along suture	**20**
19	Exocarp glabrous or glabrate, sometimes with a few hairs widely spaced over surface (49).	**49**
49(19)	Endocarp densely and uniformly short-villous; K	***Melicope cruciata***
49	Endocarp glabrous or sparsely puberulent, especially along suture	**50**
50(49)	Leaves ternate; O	***Melicope lydgatei***
50	Leaves opposite.	**51**
51(50)	Most petioles 0–10 mm long	**52**
51	Most petioles over 10 mm long	**58**
58(51)	Ovary sparsely to densely puberulent or tomentulose, but exocarp glabrate or nearly so in fruit	**59**
58	Ovary and exocarp glabrous	**60**
59(58)	Flowers perfect, carpels narrowed into a reflexed beak 10–15 mm long; WM	***Melicope oppenheimeri***
59	Flowers unisexual, carpels straight or somewhat reflexed, apex not beaked	**59a**
59a(59)	Capsules (16–)25–40(–50) mm wide, carpels connate 1/4–1/3 their length; pedicels 5–20 mm long; L, EM, H	***Melicope volcanica***
59a	Capsules 11–20 mm wide, carpels connate (1/3–)1/2 their length or more; pedicels 2–5 mm long; K, O	***Melicope wawraeana***

#### Distribution and ecology.

Although seven trees of *Melicope oppenheimeri* have been documented since its discovery in 1998, only three trees are still surviving in the upper headwaters of Waihe‘e Valley, West Maui (Figures 2, 3). No trees have yet been located outside of the type locality and access to the site has only been by helicopter.

**Figure 1.**
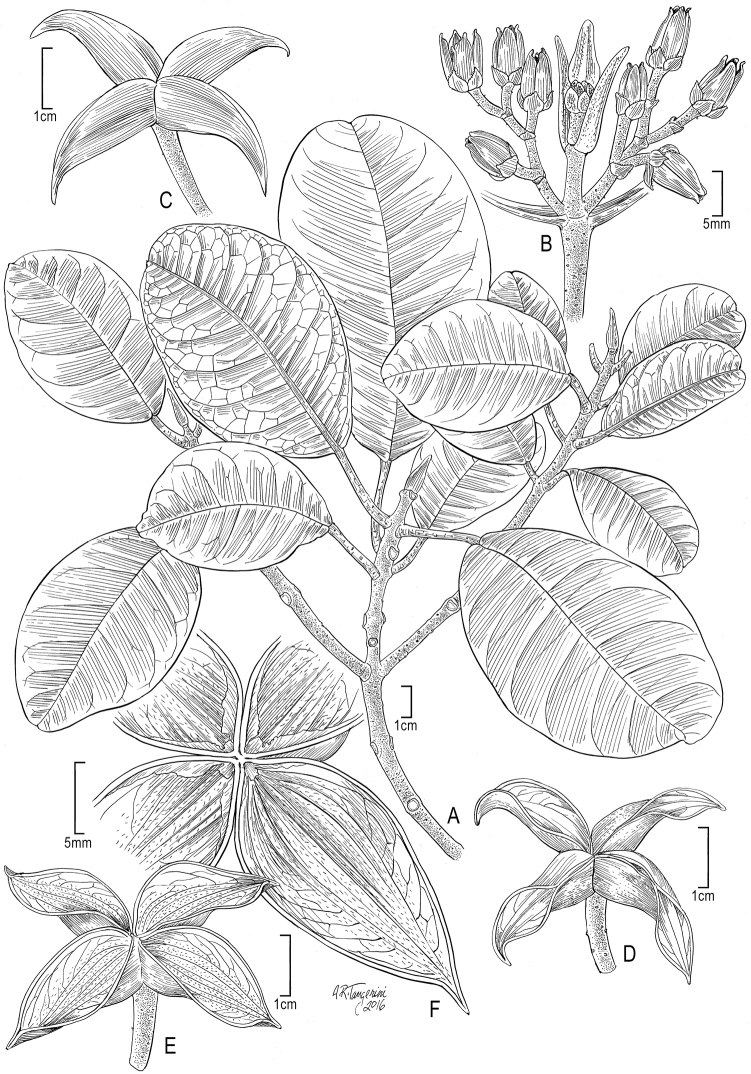
*Melicope oppenheimeri* K.R. Wood, Appelhans & W.L. Wagner. **A** Flowering branch, *Oppenheimer & Hansen H20505* (PTBG) **B** Inflorescence **C** Undehisced fruit, showing beaked carpels **D** Fruit, partly open **E** Fruit, fully opened **F** Fruit endocarp showing venation and hairs **B–F** from *Oppenheimer & Miller H90609* (PTBG) (Illustration by Alice Tangerini).

**Figure 2.**
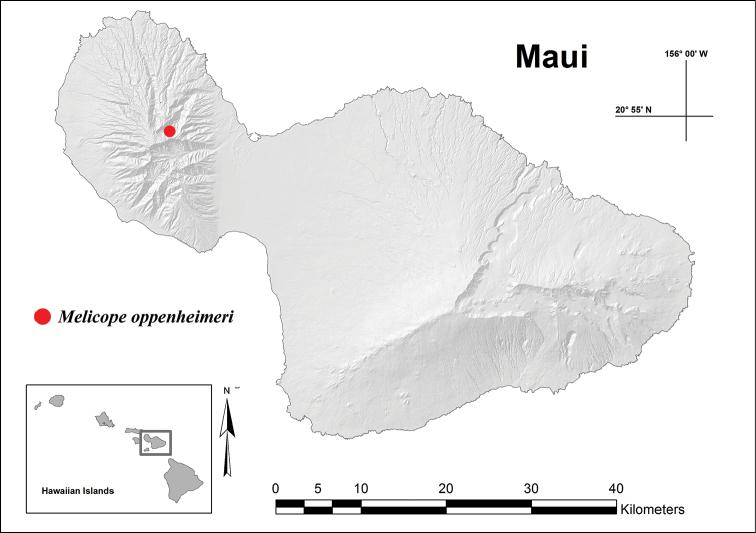
Map of Maui, Hawai‘i showing location of *Melicope oppenheimeri* in upper Waihe‘e Valley.

**Figure 3.**
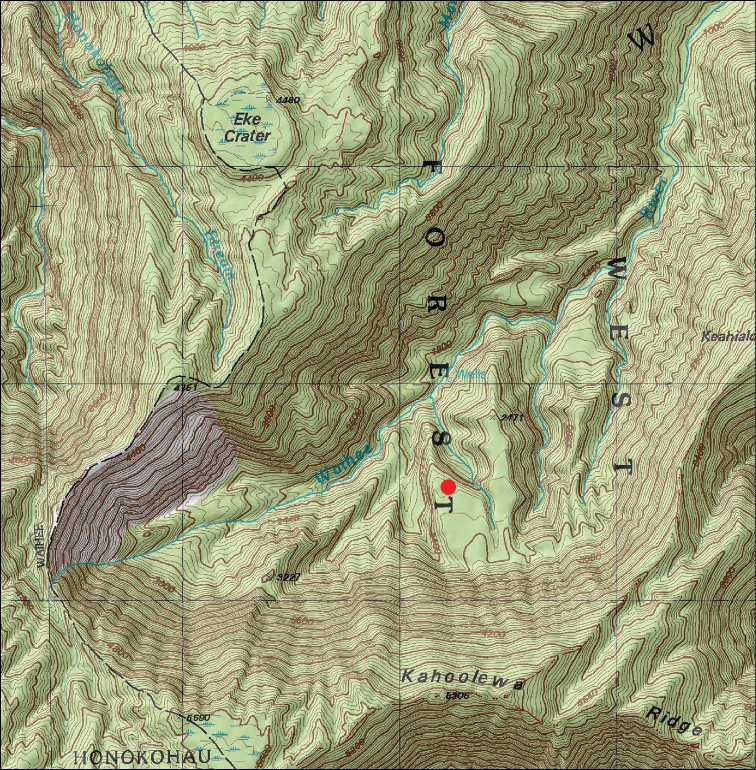
Map of upper Waihe‘e Valley, West Maui, with red dot indicating the location of *Melicope oppenheimeri* on the cliff-base plateau region.

**Figure 4.**
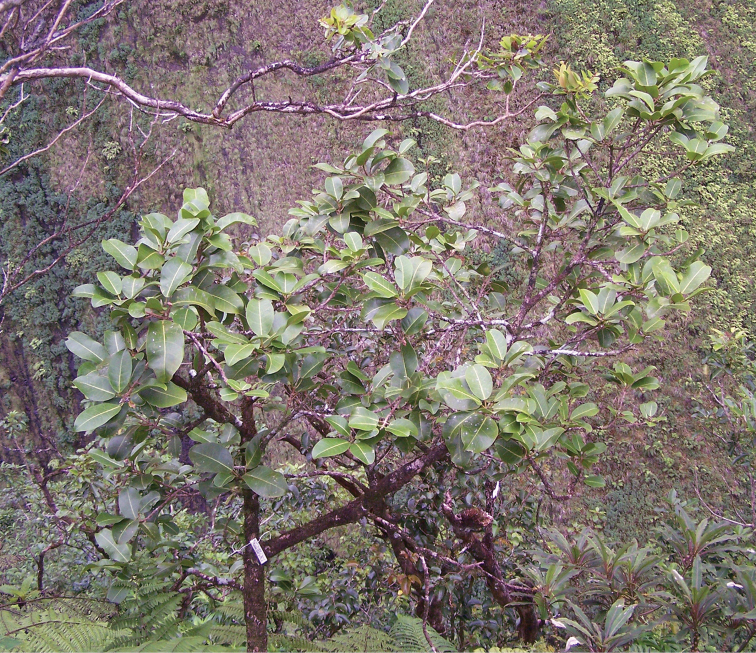
Habit of *Melicope oppenheimeri* (*Oppenheimer & Miller H90609*). Photo by H. Oppenheimer, 12 Sep 2006.

The plant communities of upper Waihe‘e Valley are varied and merge together in and around the 0.25 km^2^ cliff-base plateau region where trees of *Melicope oppenheimeri* occur. The plateau itself represents a relictual low statured *Metrosideros* Banks ex Gaertn. (Myrtaceae)-*Cheirodendron* Nutt. ex Seem. (Araliaceae) lowland wet forest community. Other relic native components of the plateau include ferns such as *Asplenium lobulatum* Mett. (Aspleniaceae), *Cibotium glaucum* (Sm.) Hook. & Arn. (Cibotiaceae), *Ctenitis latifrons* (Brack.) Copel. (Dryopteridaceae), *Diplazium sandwichianum* (C. Presl) Diels (Athyriaceae), two endemic genera of ferns, namely *Adenophorus* Gaudich. (Polypodiaceae) and *Sadleria* Kaulf. (Blechnaceae), along with herbs, shrubs, and small trees including *Antidesma platyphylla* H. Mann (Phyllanthaceae), *Broussaisia arguta* Gaudich. (Hydrangeaceae), *Coprosma pubens* A. Gray (Rubiaceae), *Dubautia plantaginea* Gaudich. (Asteraceae), *Peperomia hirtipetiola* C. DC. (Piperaceae), and various species of *Clermontia* Gaudich., *Cyanea* Gaudich. (both Campanulaceae), and *Cyrtandra* J.R. Forst. & G. Forst. (Gesneriaceae). Steeper ridges and slopes that edge the plateau are dominated by matting ferns of *Dicranopteris* Bernh.(Gleicheniaceae) (Wood 1997).

Unfortunately, past habitat degradation by introduced pigs (*Sus scrofa* L.) has altered the plant composition of the plateau, which is now being dominated by a succession of invasive weeds, which threaten *Melicope oppenheimeri*, especially *Ageratina adenophora* (Spreng.) R.M. King & H. Rob. (Asteraceae), *Buddleja asiatica* Lour. (Scrophulariaceae), *Clidemia hirta* (L.) D. Don (Melastomataceae), *Erigeron karvinskianus* DC. (Asteraceae), and *Tibouchina herbacea* (DC.) Cogn. (Melastomataceae) (Wood 1997).

Towering over the plateau and most outstanding, are vertical caldera-like basalt cliffs dominated by a native sedge and grass association called *Machaerina* Vahl (Cyperaceae)-*Deschampsia* P. Beauv. (Poaceae) wet cliff community. The cliffs are seeping with springs and waterfalls and strata of past volcanic flows are clearly evident. Additional components of this Waihe‘e cliff community include *Sadleria pallida* Hook. & Arn., *Pipturus albidus* (Hook. & Arn.) A. Gray (Urticaceae), *Eragrostis grandis* Hillebr., *Isachne distichophylla* Munro ex Hillebr. (both Poaceae), and *Dubautia scabra* (DC.) D.D. Keck.

Superb examples of *Metrosideros*-*Cheirodendron* montane wet forest dominate above these cliffs in association with windswept wet shrublands and occasional bog communities in and around the locality known as Kaho‘olewa Ridge (Wood 1997). At the base of the cliffs, which enclose the southern perimeter of the plateau lie heaps of basalt talus with accumulated substrates which are moderately deep in sections and appear to be fine textured brown silty clay.

The forests spreading below the plateau are composed of *Metrosideros* mixed lowland riparian vegetation, which are closed to open in canopy and dissected by deeply carved streams with steep banks 10–30 m in height. The native flora of this lower riparian community has similarities to the cliff-base plateau but with a greater diversity, including *Ilex anomala* Hook. & Arn. (Aquifoliaceae), *Perrottetia sandwicensis* A. Gray (Dipentodontaceae), *Polyscias oahuensis* (A. Gray) Lowry & G.M. Plunkett (Araliaceae), and *Pritchardia forbesiana* Rock (Arecaceae), along with species of *Kadua* Cham. & Schltdl. (Rubiaceae), *Myrsine* L. (Primulaceae), and *Psychotria* L. (Rubiaceae) (Wood 1997). Notable observations of native birds in the upper Waihe‘e region include nesting seabird colonies of dark-rumped petrels (*Pterodroma sandwichensis* Ridgeway) along with native forest birds such as ‘apapane (*Himatione sanguinea* Gmelin), and ‘amakihi (*Chlorodrepanis virens wilsoni* Rothschild).

#### Phylogenetic placement.

*Melicope oppenheimeri*, like all Hawaiian *Melicope*, falls into section Pelea, which has a distribution ranging from Taiwan, the Philippines, and Borneo eastward to the Hawaiian and Marquesas Islands, and south to New Caledonia. Only *Melicope triphylla* (Lam.) Merr. is distributed in the Southeast Asian areas mentioned above, and the remainder of sect. Pelea is restricted to New Guinea and Pacific archipelagos (Hartley 2001). Section Pelea proved to be polyphyletic in molecular phylogenetic analyses, and monophyly can be reached if the New Caledonian species are excluded and the Hawaiian endemic genus *Platydesma* is included in the section (Appelhans et al. 2014b). Hartley (2001, pp. 31, 139–140) stated that the Hawaiian species mostly resemble the New Caledonian species – especially *Melicope vieillardii* – based on several “primitive” characters including bisexual flowers, persistent sepals and petals, apically acute staminal filaments, basally connate carpels, glabrous endocarp, and Type A seed attachment, however, a close phylogenetic relationship of the Hawaiian and the New Caledonian species could not be verified (Appelhans et al. 2014b). The Hawaiian species of *Melicope* have been treated under the genus *Pelea* by Wagner et al. (1990) and the authors divided the taxon into the four sections *Apocarpa* B. Stone; *Cubicarpa* B. Stone; *Megacarpa* B. Stone; and *Pelea*. If the Hawaiian groups are to continue to be recognized, they would need to be treated as subsections. Only the latter of these sections, which consists of three species, proved to be monophyletic (Appelhans et al. 2014b, c). Two specimens of *Melicope oppenheimeri* (the paratypes *Wood & Perlman 7408* and *Wood & Perlman 7419*) have been included in phylogenetic research (Appelhans et al. 2014b, c) and they are listed under the original determination *Melicope reflexa* (H. St. John) T.G. Hartley & B.C. Stone in these studies. The two specimens are part of a largely unresolved clade consisting of representatives of *Cubicarpa* and *Megacarpa*. The closest relatives of *Melicope oppenheimeri* could not be determined due to the low genetic variation in the sampled nuclear and plastid markers. We are currently working on resolving phylogenetic relationships of Hawaiian *Melicope* using Next-generation sequencing.

#### Morphology and related taxa.

Although beaked fruit have evolved in a few species of *Melicope* belonging to sections *Melicope* and *Vitiflorae* (Hartley 2001, p. 19), this character is unique to *Melicope oppenheimeri* within sect. Pelea (Figure 1C, 5B). Beaked fruit have also evolved in Hawaiian Platydesma sect. Cornutia B.C. Stone, which is nested within Melicope sect. Pelea, but which is not an immediate relative of *Melicope oppenheimeri*.

**Figure 5.**
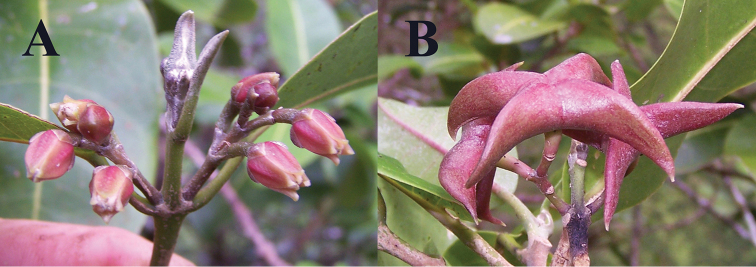
*Melicope oppenheimeri* **A** Flowers **B** Fruit, showing beaked carpels (*Oppenheimer & Miller H90609*). Photos by H. Oppenheimer, 12 Sep 2006.

Morphologically, *Melicope oppenheimeri* resembles the Moloka‘i endemic species *Melicope reflexa*, with both having reflexed carpels that are coherent at base. Significant differences between *Melicope oppenheimeri* and *Melicope reflexa* include capsules connate 1/5, 4–9 × 40–52 mm (vs. capsules connate 1/4, 10–17 × 20–33 mm); endocarp sparsely to evenly puberulent (vs. endocarp glabrous); ovary appressed densely short-sericeous (vs. ovary glabrous); pedicles 5–8 mm (vs. pedicles15–20 mm); and flowers perfect, 3–5, on robust peduncles (vs. flowers unisexual, 1–3, on delicate peduncles).

Two other morphologically similar Hawaiian *Melicope*, also with carpels coherent at base include *Melicope molokaiense* (Hillebr.) T.G. Hartley & B.C. Stone and *Melicope volcanica* (A. Gray) T.G. Hartley & B.C. Stone. *Melicope molokaiense*, which is known from Molokai and Maui, differs from *Melicope oppenheimeri* in having capsules connate 1/4, 10–17 × 21–39 mm; endocarp glabrous; ovary glabrous; and flowers unisexual. *Melicope volcanica*, which is known from Lana‘i, Moloka‘i, East Maui, and the big island of Hawai‘i, similarly differs with capsules connate 1/3–1/4, and flowers unisexual, yet also differs with leaves having 10–20 pair of secondary veins; cymes ca. 6 cm long; and peduncles 7–38 mm long. *Melicope oppenheimeri* has leaves with 7–10 pair of secondary veins; cymes ca. 3 cm long; and peduncles 5–6.5 mm long.

*Melicope* species are generally either exclusively hermaphroditic or dioecious, yet there are a few recorded exceptions (Hartley 2001, p. 10). Although *Melicope oppenheimeri* is apparently hermaphroditic, we cannot make a definitive statement on the breeding system of this rare narrow endemic, having only six collections for study, and limited flowering material.

#### Conservation status.

Plant and animal endemics from isolated oceanic islands are often endangered or critically endangered (Kreft et al. 2008, Sakai et al. 2002). The ongoing decline of native pollinators (Kearns et al. 1998) and seed dispersers (Milberg and Tyrberg 1993), in combination with other primary extrinsic factors such as invasive non-native plants, predation by introduced vertebrates, loss and fragmentation of natural habitats, and devastation by severe storms, are leading to an increase in extinctions throughout the islands of Oceania (Sakai et al. 2002, Wood 2007, 2012, Kingsford et al. 2009). Other prominent factors such as strict habitat requirements, very low historic population densities and narrow geographic range increase the risk of extinction. (Sakai et al. 2002, Wood 2007, 2015). It is currently unclear how many of the estimated 10,000 native Hawaiian insect species have gone extinct, but at this point in time the Hawaiian Islands have lost 79 of its native bird species and are left with only 32 (James and Olson 1991, Olson and James 1991, Burney et al. 2001, Boyer 2008). The authors maintain a checklist of endemic Hawaiian vascular plant taxa that have no known wild individuals remaining. Of the estimated 1191 native vascular plant species in Hawai‘i, 130 taxa are now presumed extinct. Evidently 41 of these possible extinctions have occurred in the Hawaiian lobeliads (Campanulaceae), a family renowned for their co-evolution with Hawai‘i’s unique forest birds, the honeycreepers, in the endemic subfamily Drepanidinae of the Fringillidae or finch family (Wood 2014, 2015). The Lamiaceae or mint family falls second in this severe category, with 22 species that are presumed extinct. With two-thirds of the surviving forest bird species in Hawai‘i being critically endangered and a continued decline in native arthropods, there is grave concern for the endangered Hawaiian flora and for their unique insular relationships with biodiversity as a whole. Even today, little is known about the life cycles, breeding system variations, and habitat preferences found in the Hawaiian flora (Sakai et al. 2002, Wood 2015), but it is known that *Melicope* rely on insects for pollination and birds for dispersal (Hartley 2001). Within Hawaiian *Melicope* five species are currently presumed extinct, 19 are federally listed as endangered, and with the inclusion of *Melicope oppenheimeri*, ten species fall into the Plant Extinction Prevention Program (PEPP) category, meaning there are 50 or fewer individuals remaining (see Table 1). Members of Hawaiian Rutaceae are currently in the process of being evaluated according to IUCN categories and criteria.

**Table 1.** Checklist of endemic Hawaiian *Melicope* and *Platydesma* with conservation status and island distribution. (Status Symbols: C=candidate for federal listing; E=federally listed as endangered; EX=possibly extinct; PEPP=Plant Extinction Prevention Program (50 or less individuals known in wild); SOC =species of concern. Island Distribution: K=Kaua‘i; O=O‘ahu; Mo=Moloka‘i; L=Lana‘i; M=Maui; H=Big Island of Hawai‘i; Note: *Platydesma* had been shown to be nested in *Melicope* [Harbaugh et al. 2009, Appelhans et al. 2014c]).

Taxon	Status	Distribution
*Melicope adscendens* (H.St.John & E.P.Hume) T.G.Hartley & B.C.Stone	E, PEPP	M
*Melicope anisata* (H.Mann) T.G.Hartley & B.C.Stone		K
*Melicope balloui* (Rock) T.G.Hartley & B.C.Stone	E, EX	M
*Melicope barbigera* A.Gray		K
*Melicope christophersenii* (H.St.John) T.G.Hartley & B.C.Stone	E	O
*Melicope cinerea* A.Gray	SOC	O
*Melicope clusiifolia* (A.Gray) T.G.Hartley & B.C.Stone		K, O, Mo, L, M, H
*Melicope cruciata* (A.Heller) T.G.Hartley & B.C.Stone	SOC	K
*Melicope degeneri* (B.C.Stone) T.G.Hartley & B.C.Stone	E, PEPP	K
*Melicope elliptica* (A.Gray) T.G.Hartley & B.C.Stone		O, Mo, M
*Melicope feddei* (H.Lév.) T.G.Hartley & B.C.Stone		K
*Melicope haleakalae* (B.C.Stone) T.G.Hartley & B.C.Stone	SOC	M
*Melicope haupuensis* (H.St.John) T.G.Hartley & B.C.Stone	E, PEPP	K
*Melicope hawaiensis* (Wawra) T.G.Hartley & B.C.Stone	SOC	Mo, L, M, H
*Melicope hiiakae* (B.C.Stone) T.G.Hartley & B.C.Stone	E	O
*Melicope hosakae* (H.St.John) W.L.Wagner & R.K.Shannon		O
*Melicope kaalaensis* (H.St.John) T.G.Hartley & B.C.Stone		O
*Melicope kavaiensis* (H.Mann) T.G.Hartley & B.C.Stone		K
*Melicope knudsenii* (Hillebr.) T.G.Hartley & B.C.Stone	E, PEPP	K, M
*Melicope lydgatei* (Hillebr.) T.G.Hartley & B.C.Stone	E, PEPP	O
*Melicope macropus* (Hillebr.) T.G.Hartley & B.C.Stone	EX, SOC	K
*Melicope makahae* (B.C.Stone) T.G.Hartley & B.C.Stone	E	O
*Melicope molokaiensis* (Hillebr.) T.G.Hartley & B.C.Stone		Mo, M
*Melicope mucronulata* (H.St.John) T.G.Hartley & B.C.Stone	E, PEPP	Mo, M
*Melicope munroi* (H.St.John) T.G.Hartley & B.C.Stone	E	Mo, L
*Melicope nealae* (B.C.Stone) T.G.Hartley & B.C.Stone	EX, SOC	K
*Melicope oahuensis* (H.Lév.) T.G.Hartley & B.C.Stone		O
*Melicope obovata* (H.St.John) T.G.Hartley & B.C.Stone	EX, SOC	M
*Melicope oppenheimeri* K.R.Wood, Appelhans & W.L.Wagner	PEPP	M
*Melicope orbicularis* (Hillebr.) T.G.Hartley & B.C.Stone		M
*Melicope ovalis* (H.St.John) T.G.Hartley & B.C.Stone	E	M
*Melicope ovata* (H.St.John & E.P.Hume) T.G.Hartley & B.C.Stone		K, O
*Melicope pallida* (Hillebr.) T.G.Hartley & B.C.Stone	E	K, O
*Melicope paniculata* (H.St.John) T.G.Hartley & B.C.Stone	E	K
*Melicope peduncularis* (H.Lév.) T.G.Hartley & B.C.Stone		K, O, Mo, M
*Melicope pseudoanisata* (Rock) T.G.Hartley & B.C.Stone		M, H
*Melicope puberula* (H.St.John) T.G.Hartley & B.C.Stone	E	K
*Melicope quadrangularis* (H.St.John & E.P.Hume) T.G.Hartley & B.C.Stone	E, PEPP	K
*Melicope radiata* (H.St.John) T.G.Hartley & B.C.Stone		H
*Melicope reflexa* (H.St.John) T.G.Hartley & B.C.Stone	E, PEPP	Mo
*Melicope rotundifolia* (A.Gray) T.G.Hartley & B.C.Stone		O
*Melicope saint-johnii* (E.P.Hume) T.G.Hartley & B.C.Stone	E	O
*Melicope sandwicensis* (Hook. & Arn.) T.G.Hartley & B.C.Stone	SOC	O
*Melicope sessilis* (H.Lév.) T.G.Hartley & B.C.Stone		Mo, M
*Melicope volcanica* (A.Gray) T.G.Hartley & B.C.Stone		Mo, L, M, H
*Melicope waialealae* (Wawra) T.G.Hartley & B.C.Stone		K
*Melicope wailauensis* (H.St.John) T.G.Hartley & B.C.Stone	EX, SOC	Mo
*Melicope wawraeana* (Rock) T.G.Hartley & B.C.Stone		K, O
*Melicope zahlbruckneri* (Rock) T.G.Hartley & B.C.Stone	E, PEPP	H
Platydesma cornuta Hillebr. var. cornuta	E	O
Platydesma cornuta Hillebr. var. decurrens B.C.Stone	E	O
*Platydesma remyi* (Sherff) O.Deg., I.Deg, Sherff & B.C.Stone	C, PEPP	H
*Platydesma rostrata* Hillebr.	E	K
*Platydesma spathulata* (A.Gray) B.C.Stone		K, O, M, H

*IUCN Red List Category.* When evaluated using the World Conservation Union (IUCN) criteria for endangerment (IUCN 2001), *Melicope oppenheimeri* falls into the Critically Endangered (CR) category, which designates this species as facing a very high risk of extinction in the wild. Our formal evaluation can be summarized by the following IUCN hierarchical alphanumeric numbering system of criteria and subcriteria: CR B1ab(i,ii,iii,v)+2ab(i,ii,iii,v); C2a(ii); D; which reflects a severely limited Extent of Occurrence (EOO) and Area of Occupancy (AOO) of less than 1 km^2^ and a wild population of only three individuals. It should be noted that seed collections of *Melicope oppenheimeri* have been made by Maui PEPP staff during routine monitoring and there is currently a single cultivated individual being grown at the Olinda Rare Plant Facility on East Maui.

## Supplementary Material

XML Treatment for Melicope oppenheimeri
